# Nonlocality-driven supercontinuum white light generation in plasmonic nanostructures

**DOI:** 10.1038/ncomms11497

**Published:** 2016-05-09

**Authors:** A. V. Krasavin, P. Ginzburg, G. A. Wurtz, A. V. Zayats

**Affiliations:** 1 Department of Physics, King's College London, Strand, London WC2R 2LS, UK; 2 School of Electrical Engineering, Tel Aviv University, Ramat Aviv, Tel Aviv 69978, Israel

## Abstract

Structured plasmonic metals are widely employed for achieving nonlinear functionalities at the nanoscale due to their ability to confine and enhance electromagnetic fields and strong, inherent nonlinearity. Optical nonlinearities in centrosymmetric metals are dominated by conduction electron dynamics, which at the nanoscale can be significantly affected by the nonlocal effects. Here we show that nonlocal corrections, being usually small in the linear optical response, define nonlinear properties of plasmonic nanostructures. Using a full non-perturbative time-domain hydrodynamic description of electron plasma under femtosecond excitation, we numerically investigate harmonic generation in metallic Archimedean nanospirals, revealing the interplay between geometric and nonlocal effects. The quantum pressure term in the nonlinear hydrodynamic model results in the emergence of fractional nonlinear harmonics leading to broadband coherent white-light generation. The described effects present a novel class of nonlinear phenomena in metallic nanostructures determined by nonlocality of the electron response.

## Abstract (web summary)

A theoretical description of the nonlinear response of metals is complicated due to their complex electron behavior. Here, Krasavin *et al*. use a hydrodynamic model coupled with Maxwell's equations and demonstrate higher harmonics and supercontinuum generation from metal Archimedean spiral shapes

## Graphical abstract

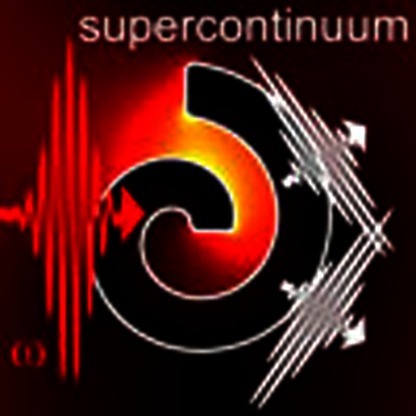

Nanostructured electromagnetic environments enable tailoring and manipulation of optical interactions on subwavelength scales^1^. The introduction of high refractive index dielectrics and plasmonic metals, such as gold, silver and recently developed semiconductor-based compounds^2^ enable achieving subwavelength confinement^3^ and, as a result, increase the strength of light–matter interactions. Particularly, metallic nanostructures support plasmonic modes, which at a certain frequency, come in resonance with the incident field and drastically increase the intensity of the local field with extremely small mode volumes. Generally, nonlinear interactions depend on the local field intensity at powers predefined by the order of the interaction (for example, a power of two for second harmonic generation (SHG) which is a second-order nonlinear process). This power law indicates the advantage of the field enhancement and modal volume reduction for enhancing strengths of nonlinear interactions. The majority of experimental demonstrations, utilizing this approach, rely on strong local field enhancement, delivered by plasmonic structures in the vicinity of nonlinear materials of different kind, for example, polymers^4^, noble gasses^5^ and others (reviewed in ref. 6).

However, metal composites themselves are strongly nonlinear media and capable of generating nonlinear harmonics. At the same time, since the permittivity of noble metals in visible and infra-red spectral range is negative, electromagnetic waves cannot propagate inside the bulk material where only evanescent components are supported, minimizing the overlap between the field and nonlinear material, and consequently reducing the efficiency of the overall response. Nevertheless, nanostructures with features smaller than the skin depth will allow the field to penetrate into the metal thus paving the way for tailoring their linear^7^ properties and maximize their nonlinear response by designed structuring^8^.

A theoretical description of inherent nonlinear responses of metals is a fairly complicated task due to the natural complexity of their solid state structure. Along with phenomenological models, relying on experimental retrieval of nonlinear susceptibilities, a hydrodynamic model, treating the electron plasma by means of a charged fluid, was shown to give a qualitative description of the nonlinear interaction. In particular, predictions of second-order responses^9^,^10^ and Kerr-type nonlinearities^11^ qualitatively agree with existent experiments, obtained for pump wavelengths away from interband transitions. A hydrodynamic model with additional Lorentzian resonances terms is capable to reproduce metal susceptibilities over the entire spectral range^12^. Furthermore, the mesoscopic hydrodynamic approach is integrable with electromagnetic modelling, enabling studies of large scale electromagnetic systems with nontrivial geometries. It is worth noting that *ab initio* microscopic models are hardly extendable beyond bulk and flat surfaces descriptions due to enormous computation complexity involved^13^.

Here, using a fully non-perturbative time-domain hydrodynamic model coupled with Maxwell's equations, we demonstrate higher harmonics and supercontinuum generation from metal nanostructures of Archimedean spiral shapes. Properly tuned geometry with the lack of any symmetry (both rotational and reflection) maximizes the nonlinear response and shows the appearance of the 6th and higher harmonics, which was neither theoretically analysed nor experimentally observed to date. The robustness of the approach is evident from the fact that a non-perturbative time-domain hydrodynamic description in spherical/cylindrical geometries reliably reproduced experimental data^14^, allowing to extend this method for addressing novel physical scenarios. Being able to investigate the interplay between the topology of the nanostructure and various sources of nonlinearities of metal plasma, we demonstrate that electromagnetic nonlocality, manifesting itself via the quantum pressure term, has the prominent role in the nonlinear response of small and nanostructured (on ∼10 nm length scales) geometries. The properly tuned interplay between nonlocality and nonlinearity is responsible for very efficient harmonics mixing and the resulting broadband white light generation, as demonstrated below.

## Results

### Coupled electromagnetic-hydrodynamic nonlinearities

The interaction of electromagnetic waves with material bodies can be described via an induced polarization (**P**(**r**,*t*)) inside the latter. In the time domain, the interaction dynamics in the case of nonmagnetic structured media is given by


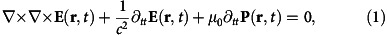


where **E**(**r**,*t*) is the electric field, *c* is the speed of light in vacuum and *μ*_0_ is the vacuum permeability. In general, the spatio-temporal polarizability holds all the information on both linear and nonlinear responses of the material, also including the chromatic dispersion. Nonlocality will enter the expression through spatial derivative terms in the real coordinate's space. The polarizability of metal structures can be introduced in this equation via natural hydrodynamic variables: the macroscopic position-dependent electron density *n*(**r**,*t*) and velocity **υ**(**r**,*t*), which are subsequently related to the polarization current as


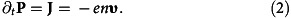


On the other hand, the dynamics of the free electron gas is determined by a set of hydrodynamic equations^10^


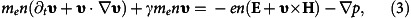



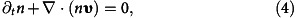


where *m*_*e*_ and *e* are the electron mass and charge, respectively, *γ* is the effective scattering rate, representing optical losses in a phenomenological way, and 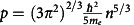 is the quantum pressure term evaluated within the Thomas–Fermi theory of an ideal fermionic gas. Equations (1, 2, 3, 4) are inherently nonlinear and provide a self-consistent formulation of nonlinear optical processes originating from free conduction electrons in plasmonic systems. In the perturbative regime of nonlinear interaction (weak pump field approximation), the second-order nonlinear polarisation plays the leading role, resulting from the convective acceleration term 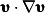, the magnetic component of the Lorentz force 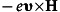, and *n***υ** term (ref. 9). The quantum pressure term was shown to have minor contribution in 100-nm-size geometries^14^. The further increase of the peak power of the pump pulse brings higher-order nonlinear terms into consideration, resulting in intermixing of bulk and surface nonlinear effects. It is worth noting that high harmonics (higher than 3rd) with the metal clearly identified as the nonlinear source were not yet reported. Recently, attosecond pulse generation from metallic structures were predicted by applying ultra-strong electromagnetic fields, changing the quantum wavefunctions of electrons^15^.

Another very important feature of the hydrodynamic description is its inherent ability to describe nonlocal electromagnetic effects. Generally, nonlocality is the result of strong coupling between adjacent unit cells in a material, either in natural crystals or artificial materials (metamaterials), many body effects in solid state systems, and others (for example, ref. 16). Hydrodynamic nonlocality is the typical example of strong electron–electron interactions between quasi-free electrons of the metal plasma and was proven to describe a variety of phenomena, governing the optical response of small plasmonic structures^17^. In the linear optics regime, the quantum pressure term is the one responsible for the appearance of nonlocal effects, as it contains a spatial derivative in the linearized model due to the presence of the fractional power (5/3) in the electron density^18^. However, the interplay of nonlocality and nonlinearity was neither considered nor investigated before.

### Non-perturbative time-domain numerical model

**Figure 1:**
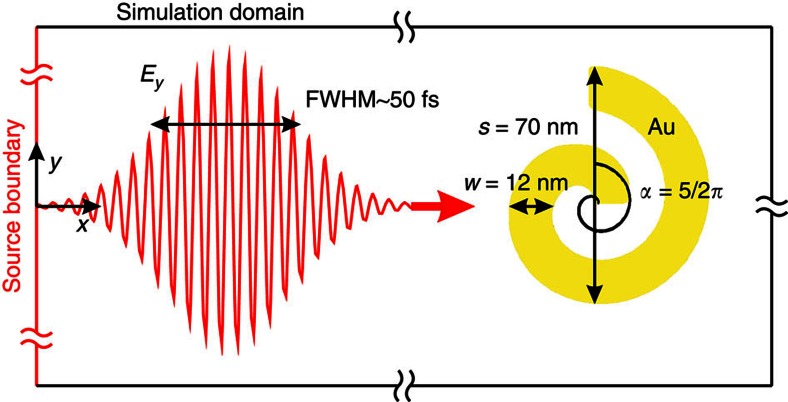
Layout of the time-domain numerical simulations. In the finite element time-domain numerical simulations, a ∼50 fs vertically polarized optical pulse with a central wavelength of 1,500 nm is generated at the domain source boundary and illuminates an Archimedean nanospiral from the left. Geometrical parameters of a nanospiral are also shown.

For a comprehensive analysis of nonlinear interactions from nanostructures, the set of equations (1, 2, 3, 4) was implemented with the help of a finite-element time-domain method. Two-dimensional geometries under transverse magnetic field illumination with driving electric field polarized across the nanostructure (Fig. 1) were considered to reduce the computational complexity (electric field has only in-plane components, while magnetic field is directed out of plane). In such configuration, plasmonic resonances in small metal structures may be studied without requiring the presence of the third dimension, which introduces just a geometrical correction factor. A Gaussian pulse was specified at the source boundary of the simulation domain with a spatio-temporal profile 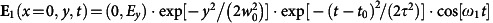, where the fundamental frequency *ω*_1_=1.257 × 10^15^ s^−1^ corresponds to a free-space wavelength of *λ*_1_=1,500 nm, the temporal width *τ*=20 fs (FWHM ∼47 fs) and the spatial width *w*_0_=*λ*_1_/2 (Fig. 1). The resulting propagating pulse has a linear polarization in the *y* direction and is incident on the nanostructures along the *x* direction. Fundamental light intensities of up to *I*_0_=1.8 × 10^18^ W m^−2^ were considered which the nanostructure can still withstand^19^.

### The role of quantum pressure in nonlinear response

**Figure 2:**
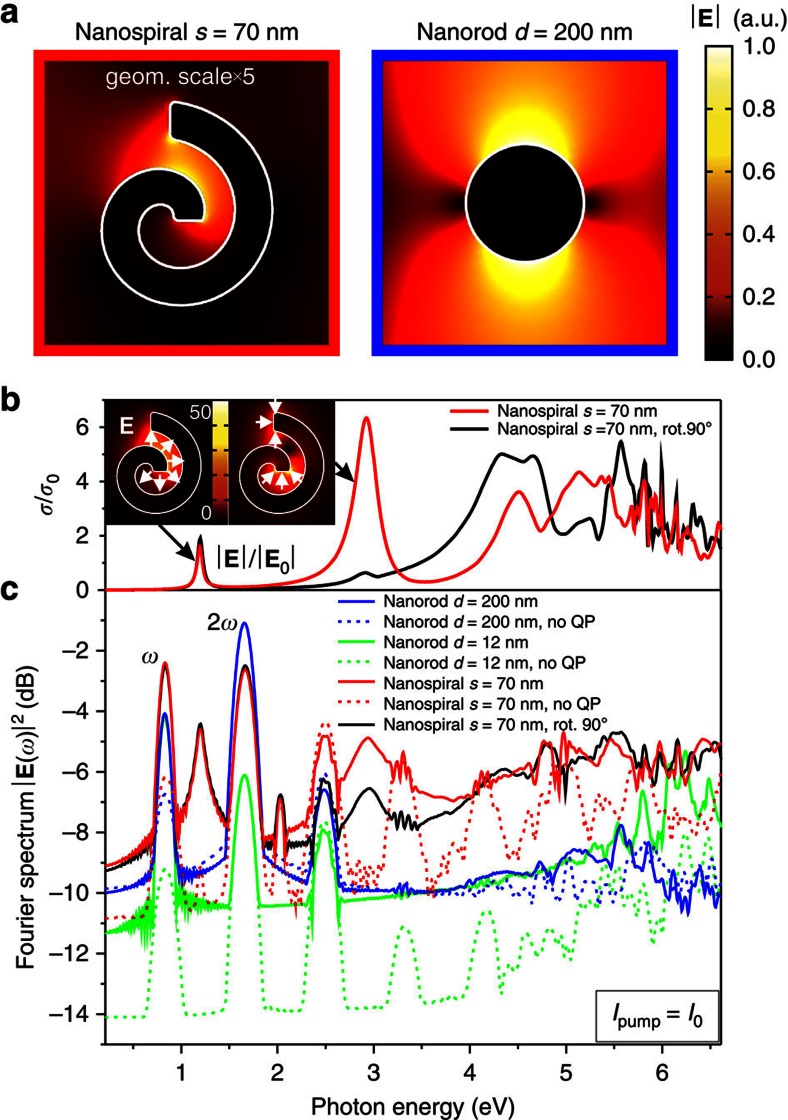
Nonlinear response of Archimedean nanospiral. (**a**) Electric near-field maps around the metallic nanospiral with *s*=70 nm and *w*=12 nm and the nanorod with *d*=200 nm. (**b**) Extinction cross-section spectra of the nanospiral normalized to its geometrical area modelled with linear frequency-domain numerical simulations. The insets show the electric field enhancement maps 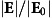 for the first two nanospiral resonances with respect to the norm of the electric field of the incident wave. The white arrows show the direction of the local electric field. (**c**) Nonlinear scattering spectra of the nanospirals and nanorods of different parameters for an excitation pulse with *τ*=20 fs: solid and dashed lines correspond to the hydrodynamic model with and without the quantum pressure term, respectively.

To investigate the effect of the nanostructure's geometry on nonlinear generation, we first compare the performance of a perfectly symmetric cylindrical nanorods (diameters *d*=200 and 12 nm) with an Archimedean spiral shape nanostructure (spiral angle *α*=5/2*π*, arm width *w*=12 nm, overall size *s*=70 nm (Fig. 2a,b). Spirals have no symmetry of any kind and, hence, are good candidates for nonlinear optical interactions as they are not obeying any geometrical selection rules^20^. Initially, for fair comparison of nonlinear responses of different shapes, we consider a nonresonant excitation when the excitation frequency is lower than the lowest plasmonic resonances of both nanorods and nanospirals.

The unique ability to either include or exclude the quantum pressure term in the numerical model enables investigations of the impact of nonlocality on the nonlinear generation. For large cylinders of 200 nm diameters (blue solid and dashed lines in Fig. 2c), the nonlinear scattering intensity (with linear scattering field subtracted) shows a clear signature of higher harmonics up to the 3rd order, though no significant impact of the nonlocality. For smaller cylinders of 12 nm in size, the role of the quantum pressure is more significant. At the length scale of few nm (*r*=6 nm in our case), which is smaller than the mean free pass of electrons and becomes comparable with the radius of nonlocality related to the electron Fermi wavelength (∼0.5 nm), the nonlocal response starts playing an important role in the nonlinear scattering of the nanostructures. While the structure of the local and nonlocal spectra remains almost unchanged up to the 3rd harmonic (dashed and solid green lines in Fig. 2c, respectively), the generated intensity between integer harmonics in the nonlocal case is tremendously enhanced compared with the local counterpart, indicating to the presence of fractional harmonics. The effect of nonlocality, however, is much more pronounced in the case of the spiral nanostructure (red lines in Fig. 2c). First, the appearance of fractional harmonics, due to the quantum pressure term, is evident. A pronounced and remarkable difference between local and nonlocal scenarios manifests itself in the broadband supercontinuum generation. It originates from collective electron–electron interactions giving rise to distinct fractional harmonics (due to the power of 5/3 in the quantum pressure term in Eq. (3)), which provide an efficient pathway for enhancing frequency mixing between natural integer harmonics. For example, in conventionally used nonlinear materials for supercontinuum generation, broad white-light spectrum is achieved via higher-order interactions of the finite width peaks, centred at around integer multiples of the fundamental frequency. Consequently, the appearance of fractional harmonics relaxes the demand for multiple interactions, making the non phase-matched nanoscale process extremely efficient. It is worth noting also, that the efficiency of nonlinear generation from the spiral structure is substantially higher than that of cylinders.

The second factor underlying efficient supercontinuum generation is the abundance of high-frequency modes (Fig. 2b) available for coupling of the light generated through nonlinearity, which in turn enhances far-field radiated light due to the so-called double-resonant regime^8^. This is evident on the example of two lowest resonances of the nanospiral at 1.2 and 2.9 eV (Fig. 2b) showing a prominent nonlinear signal increase at their frequencies including the spectral range between the first and second harmonics (cf. red lines in Fig. 2b,c). If the nanospiral is illuminated from the different side (rotated 90° clockwise, Fig. 2b), the second resonance is excited via nonlinear response much less efficiently, which leads to the decrease of the corresponding nonlinear signal (cf. red and black curves in Fig. 2c at the frequency of 3 eV). Judging from the intensity distributions and field orientation (white arrows in the insets in Fig. 2b), this resonance corresponds to the excitation of the nanospiral with the field concentration at the extremities of the spiral. The lowest resonance is due to electromagnetic coupling of turns of the spiral, similar to modes of metal-dielectric-metal waveguides^21^. As a concluding remark, we note that the linear spectra of the nanospiral illuminated from opposite sides are very similar, while controlling direction of illumination allows to control the relative contribution of difference resonances (Fig. 2b).

### Resonant coherent nonlinear response

The plasmonic resonances of the spiral (Fig. 2b) can be easily tuned to the required wavelength by changing geometrical parameters of the spiral, boosting the intensity of the nonlinear effects via the field enhancement associated with coupling of the excitation light to plasmonic resonances. Resonant properties of plasmonic nanostructures give them decisive advantage for generation high-intensity nonlinear signals at the nanoscale. When the frequency of the excitation light matches the frequency of the plasmonic mode, the latter is resonantly excited, which leads to pronounced enhancement of the local fields. The enhancement of the associated nonlinear signal is even more dramatic, since its intensity is proportional to higher powers of the excitation intensity, given by the order of the interaction. From this point of view, nanospirals present a very robust and efficient class of nanostructures, offering well-defined narrow resonances, which can be easily adjusted to a given frequency.

**Figure 3:**
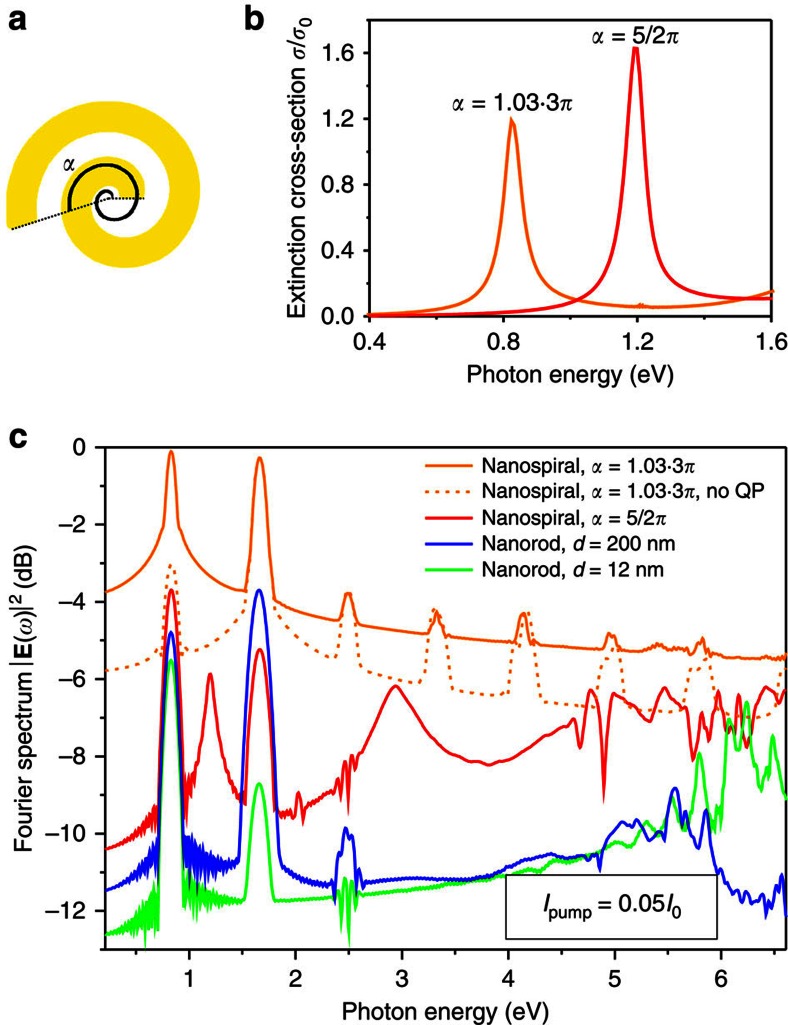
Resonant nonlinear response. (**a**) Schematic of the nanospiral indicating the angle *α*, defining the positions of the nanospiral resonances. (**b**) Spectra of the extinction cross-sections of the nanospirals with *α*=5/2*π* and *α*=1.03·3*π* normalized to their geometrical area (*s*=70 nm and *w*=12 nm). (**c**) Nonlinear scattering spectra of the nanospirals with *α*=1.03·3*π* (the resonance at the fundamental frequency) and *α*=5/2*π* and the nanorods with *d*=200 nm and *d*=12 nm.

In the case of spiral parameters considered above, the local fields are enhanced by factors of ∼35 and 50 in the first (1.2 eV) and second (2.9 eV) plasmonic resonances, respectively (Fig. 2b). Taking the advantage of flexibility of the geometry, the lowest plasmonic resonance of the nanospiral was matched to the frequency of the pump (1,550 nm or 0.83 eV) by increasing the angle of the spiral (and consequently its length) to the value *α*=1.03·3*π* slightly above 3*π* (Fig. 3a,b). This optimized structure provides the highest field enhancement in the first resonance, rather than the second one.

**Figure 4:**
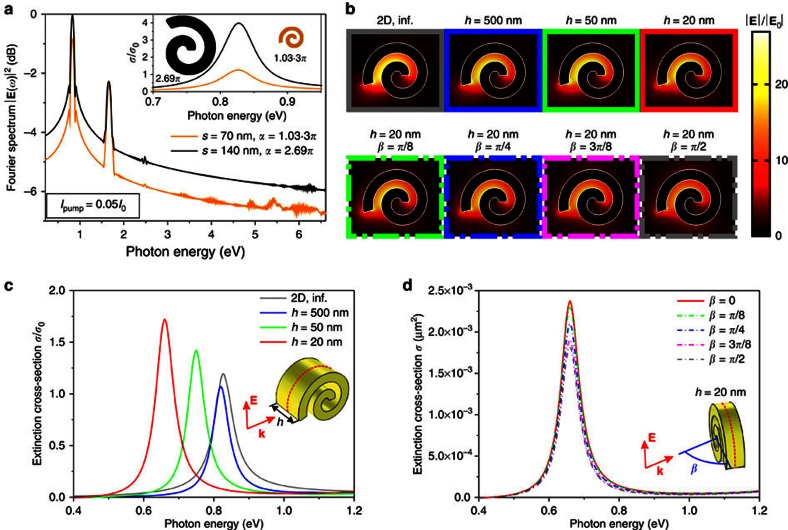
Dependence of the resonant properties on nanospiral geometric parameters. (**a**) Nonlinear scattering spectra of the nanospirals resonant at the fundamental frequency for the designs with *s*=70 nm, *w*=12 nm, and *α*=1.03·3*π* (orange line) and *s*=140 nm, *w*=24 nm, and *α*=2.69*π* (black line). (**b**) Electric field distributions 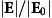 at the fundamental nanospiral resonance for (top) different nanospiral thicknesses at normal incidence (the spectra are shown in **c**) and (bottom) different incident angles for nanospiral thickness *h*=20 nm (the spectra are shown in **d**). The field maps were taken across the nanospiral midplane marked in the insets in **c** and **d** by a dashed red curve. (**c**) Dependence of the nanospiral extinction spectra on the thickness at normal incidence. (**d**) Dependence of the extinction spectra of a 20-nm-thick nanospiral on the angle of incidence. The other nanospiral parameters in **b**–**d** are *s*=70 nm, *w*=12 nm, and *α*=1.03·3*π*.

As one can see by comparing the nonlinear responses of the resonant spiral to the nonresonant spiral and the nanorod (*d*=200 nm), the intensity of the SHG signal increased by >5 and 4 orders of magnitude, respectively, with further several orders of magnitude increase throughout the nonlinear supercontinuum spectrum (Fig. 3c). Comparison with the nonlinear spectrum of a nanorod with a diameter equal to the nanospiral arm width (*d*=12 nm), the difference is even more striking with more than 9 orders of magnitude increase of the SHG intensity and 8 to 3 orders of magnitude increase of higher-harmonic intensities. Such striking difference highlights the importance of the resonant effects for enhancing the nonlinear interactions as well as the importance of topology of the nanostructure: the surface areas and volumes of these two structures are different by only one order of magnitude: 10 and 20 times, respectively. An effective second-order nonlinear susceptibility *χ*^(2)^=600 pm V^−1^ of a nanospiral can be estimated considering a material with *χ*^(2)^ that produces the same overall SHG flux. This effective susceptibility is 20 times higher than that of lithium niobate^22^. This value for the simulated nanostructures in the absence of nonlocal effects is consistent with the experimentally observed values of the effective second-order susceptibility of metallic nanostructures of 10 pm V^−1^ (ref. 20) and 3.2 pm V^−1^ (ref. 23), taking into account the local field enhancements and the surface areas. The SHG enhancement is robust with respect to geometrical scaling under the resonant excitation of the fundamental nanospiral mode which size-dependent spectral position can be matched to the fundamental wavelength by varying *α* (Fig. 4a). The SHG intensity from the twice larger nanospiral increases by the factor of ∼1.4 in accordance with a similar increase of the surface area. In the nonresonant case of the nanorods much smaller than the wavelength and without nonlocality, the SHG intensity scales with size as 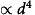 (ref. 24), which is in excellent agreement with 5 orders of magnitude difference in the SHG intensities from 12 and 200 nm nanorods (dashed lines in Fig. 2). Such behaviour is the result of much smaller dipolar (which is retardation-related) and quadrupolar moments excited at the SH frequency for a smaller nanorod.

To demonstrate robustness of the effect for a wide range of experimentally relevant scenarios, the three-dimensional spirals of different thicknesses under various illumination conditions were simulated (Fig. 4b–d). The high-Q factor nanospiral resonance, a key to the observed high nonlinear susceptibilities, was confirmed for the nanospiral thickness *h* ranging from infinity (two-dimensional case considered above) down to the deep-subwavelength thicknesses (Fig. 4c). With the decrease of the thickness of the spiral, the fundamental resonance experiences a red shift. However, the nature of the resonance, as can be seen in the field maps in Fig. 4b, remains the same with the similar field distributions and field enhancement values. Thus, by adjusting the angle *α*, the resonance position can be kept at the same fundamental wavelength for spirals of different finite thicknesses, so that the efficiency of the nonlinear processes is similar. Furthermore, this behaviour remains the same when the light is obliquely incident at a nanospiral (Fig. 4b,d).

## Discussion

The interplay between nonlocality and nonlinearity on metal nanostructures was investigated. It was shown that the quantum pressure, the manifestation of collective many body dynamics of electron plasma in metals, together with structure topology plays the key role in the process of nonlinear harmonic generation. The appearance of high harmonics (up to 6th) and broadband white light generation from spiral shaped nanostructures is the result of the interplay between local geometry and fractional harmonic generation by the nonlocal quantum pressure term. Fractional harmonics were shown to mix efficiently with natural integer ones via nonlinear interaction processes to give rise to strong supercontinuum generation. The interplay between integer and fractional harmonics is unique for plasmonic systems, favouring them over existent nonlinear materials particularly on the nanoscale.

Coupled nonlinearities, macroscopic and microscopic effects (nonlinear mesoscopic phenomena), being one of the hardest tasks for analytical and numerical solutions, have been solved here for the first time. The implemented semi-phenomenological method enables addressing basic collective and, as a result, nonlocal effects of the electron plasma and is applicable in a range of validity of the hydrodynamic model. The latter neglects a number of quantum and classical phenomena, such as the electron spill out, tunnelling across small gaps, temperature effects, ultra-fast nonequilibrium dynamics and few others. Nevertheless, the majority of the effects, beyond the scope of the model, provide higher-order corrections. As a result, the hydrodynamic model is known to provide qualitative results in a good agreement with the majority of experimental observations. Its integrability with large scale, geometry-invariant structures makes the approach a universal tool of nonlinear analysis. From the application stand point, the developed approach provides a guideline for designing nanoscale nonlinear devices important in modern photonic technologies.

## Methods

### Modelling of the transient nonlinear optical response

The nonlinear optical response of the plasmonic nanostructures was studied using time-domain finite element method simulation of an electromagnetic problem defined by a set of Maxwell's equations coupled in a self-consistent way to additional partial differential equations implementing the hydrodynamic model in a framework of Comsol Multiphysics software. The Maxwell's equations were expressed in terms of a vector potential **A**


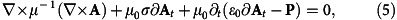


while the hydrodynamic description of nonlinear transient response of plasmonic nanostructures given by equations 2, 3, 4 was introduced through coefficient form (equations (2) and (3)) and general form (equation (4)) of partial differential equations. The material for nanostructures was chosen to be gold. The constants for gold permittivity were taken to be *n*_0_=5.98 × 10^28^ m^−3^, *γ*=1.075 × 10^14^ s^−1^, *ω*_p_=13.8 × 10^15^ s^−1^, relying on available and widely used tabulated data. The simulation domain size was set to be 6 × 6 μm^2^ to ensure that the outer domain boundaries had no effect on the simulation results, which was checked. The simulation time span *T*=7*τ* and offset *t*_0_=3*τ* were chosen so that both pumping and scattered light pulses (the latter containing higher harmonics) entirely propagated across the simulation domain, ensuring the complete and time-interval-independent modelling of nonlinear effects.

### Modelling of resonant properties of plasmonic nanostructures

The spectral response of the plasmonic nanostructures was simulated using frequency-domain finite element method in scattered-field formulation. The size of the simulation domain and the linear optical parameters of gold were set to be consistent with the time-domain simulations. The nanostructures were illuminated with a plane wave with parametrically changed frequency, while perfectly matched layers were implemented around the simulation domain to ensure the absence of reflection of the scattered waves from the outer boundaries. The nanostructure's extinction spectrum was calculated through the sum of the scattering and absorption cross-sections. The latter two parameters were calculated via integrating the incoming total power flow (for the absorption) and outcoming scattered power flow (for the spattering) over a nanoscale cylindrical region around the nanostructure and normalizing the obtained value to the power flow incident on the nanostructure's geometrical cross-section.

## Additional information

**How to cite this article**: Krasavin, A. V. *et al*. Nonlocality-driven supercontinuum white light generation in plasmonic nanostructures. *Nat. Commun.* 7:11497 doi: 10.1038/ncomms11497 (2016).
